# Exploring the Determinants of Perceived Risk of Middle East Respiratory Syndrome (MERS) in Korea

**DOI:** 10.3390/ijerph15061168

**Published:** 2018-06-04

**Authors:** Sunhee Kim, Seoyong Kim

**Affiliations:** 1 Department of Public Administration, Seowon University, Musimseoro, Cheongju 28674, Chungbuk 361-742, Korea; shkim7675@hanmail.net; 2 Department of Public Administration, Ajou University, Worldcup-ro, Suwon 16499, Korea

**Keywords:** risk, risk perception, risk communication, value, resource, Middle East Respiratory Syndrome

## Abstract

The world is turning into a risky society. Although modernization based on the developments in science and technology has increased individuals’ well-being and wealth, the perceived risk toward the complex technological system has increased. In a risky society, social accidents amplify the existing fear among individuals. It is generally assumed that each value, perception, and resource influences the fear of risk. However, very few studies have tested these three factors together within an integrated causal model. Therefore, the present study aimed to examine the determinants that influence the perceived risk in cases of Middle East Respiratory Syndrome (MERS), a deadly epidemic disease, in Korea. Based on the theoretical model, we analyzed the survey data collected from respondents (*N* = 814) in Korea. After controlling for variables such as sociodemographic characteristics, we examined how three competing factors, i.e., value, perception, and resource, influence the perceived risk of MERS. The analysis showed that trust and vulnerability variables in the perception factor, health state, and perceived knowledge in the resource factor had a significant impact on the perceived risk of MERS.

## 1. Introduction

**Figure 1:**
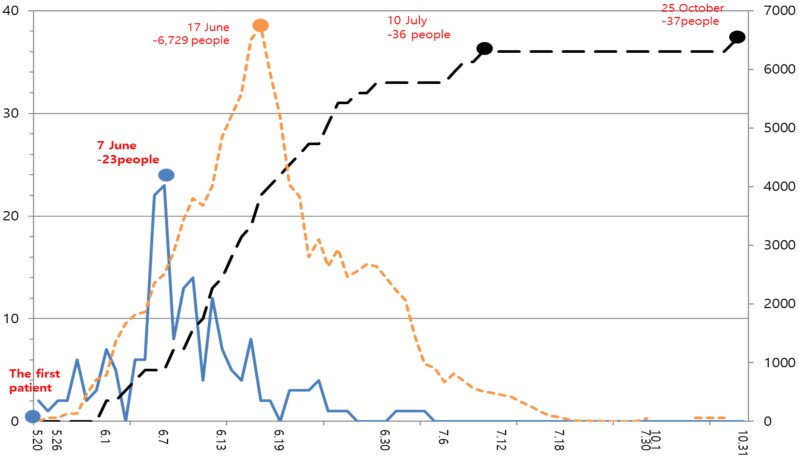
Statistics on the incidence of Middle East Respiratory Syndrome. Note: black line, the number of deaths; dark blue dotted line, the number of confirmed cases with MERS; light yellow dotted line, the number of quarantined cases. Data source: Seoul metropolitan government [1].

In 2015 when the Middle East Respiratory Syndrome (MERS) epidemic broke out in Korea, 186 individuals contracted the disease, 38 died, and 16,752 were quarantined. This serious epidemic created the biggest social fear in Korea. The first MERS case was identified on 20 May 2015; a 68-year-old man returning from the Middle East was diagnosed with MERS. It passed nine days after he had sought medical care. Figure 1 shows repaid spread of MERS. In Figure 1, the X axis represents the timeline. The patient with MERS was first confirmed on 20 May 2015. The Y axis presents the number of deaths (black line), the number of confirmed cases with MERS (dark blue dotted line), and the number of quarantined cases (light yellow dotted line). The number of quarantined individuals peaked on 17 June, with 6729 individuals. The number of death caused by MERS peaked on 25 October, with 37 cases. On 7 June, 23 persons were confirmed with MERS.

MERS influenced not only the society but also the economic state of Korea. Amid the rapid spread of MERS, according to CNN International, (17 June 2015 in South Korea) as of 17 June 2015, the reservation of 100,000 tourist visits to the nation had been cancelled. Furthermore, according to the Minister of Strategy and Finance, South Korea’s department store sales had decreased by 16.5% as compared to the same period before 2015, and sales in retail stores also decreased by 3.4%.

Risk perception toward a specific object or event may be based not only on objective or physical factors, e.g., large number of deaths, but also on subjective or constructive ones, e.g., the perceived risk from the death of one beloved person. Therefore, risk judgment is considered to be a social phenomenon. In terms of subjective views, the psychometric paradigm in risk studies suggests that lack of control, high catastrophic potential, and severe consequences account for increased risk perception and anxiety associated with dread risks [2]. With reference to MERS, Yang and Cho [3] argued that the MERS outbreak in Korea was exacerbated by inappropriate responses by major institutions. They explained that the Korean government did not deliver timely information about the status of the epidemic, hospitals’ names, and response procedures related to MERS. In particular, not disclosing the information of hospitals that accommodated patients with MERS brought about unnecessary anxiety and fear among the public. Instead of the government, the Pressian, a press media organization, was the first to announce the list of hospitals accommodating MERS patients. Under such circumstances of limited information, individuals tend to depend on rumors or informal information. In particular, the public obtained inaccurate information from social media and the internet. Such unfiltered information increased the level of perceived risk of MERS, all of which amplified the dread related to this disease. Unfiltered information is closely related to rumors. Rumors amplify the perceived risk. For example, according to Jacob et al. [4], rumors related to the Fukushima nuclear accident rapidly spread across the country border, and, once spread, they exacerbated the disaster situation. In the case of MERS, rumors such as “Several cases were confirmed at hospitals in Suwon and Pyeongtaek, Gyeonggi Province, and the disease has been already transmitted to a number of individuals there”, “Foreign media reports that Korea is under disaster, saying MERS will become a more serious epidemic than Ebola or SARS”, were spread [5]. However, these rumors were not true. The lack of credible information is often filled by rumors.

MERS has changed individuals’ attitudes toward the health system. For example, Al Ghobain et al. [6] emphasized that MERS outbreaks have had a negative psychological impact on health care systems in Saudi Arabia. Their study tested the psychological impact of Middle East Respiratory Syndrome CoronaVirus (MERS-CoV) outbreaks on emergency room resident physicians (ERRPs). Among the ERRP survey respondents, majority (91%) agreed that their work put them at risk of infection. A total of 54% of ERRPs reported being afraid of contracting the infection from infected patients. Further, a majority of the ERRPs (85%) felt that their job would expose their families to risk of infection [6]. Lee et al. [7] investigated relationships between community residents’ infection sensitivity and their levels of preventive behaviors during the 2015 MERS outbreak in Korea. They demonstrated that the group with overall high sensitivity exhibited higher scores on stress levels, reliability on preventive behaviors, practice of preventive behaviors, and practice of hand washing during the outbreak period, as compared to the non-sensitive group.

Even though several studies related to MERS have been conducted in Korea, few suggest and verify a more integrated causal model that includes various factors that influence the perceived risk of MERS. Therefore, the present study aims to identify determinants that have a significant impact on the perceived risk of MERS. Although three competing factors, i.e., value, perception, and resources, have frequently been discussed as determinants of risk perception, very few studies have integrated these three factors in a research model. Therefore, we proposed and tested a more comprehensive model comprising these three factors.

## 2. Theoretical Background and Hypothesis

### 2.1. Previous Studies about MERS

Several previous studies have focused on the “media effect” of MERS or “public response” to MERS. For example, after assessing the risk perception and practices of 78 camel owners by using the interview method, Makhlouf et al. [8] showed that most of the owners were aware of the disease (93.6%), mostly as a result of TV (75.6%). Song et al. [9] investigated online diffusion of information, spread of fear, and perceived risk of MERS infection by examining 8,671,695 MERS-related online documents from 20 May to 18 June 2015, retrieved from 171 Korean online channels. They reported that a buzz of negative emotions (i.e., anxiety or fear) was more prevalent in online discussion boards, on Twitter, and in online cafes, rather than on news sites and blogs. Additionally, news buzz, but not rumor buzz, was associated with positive MERS emotions (i.e., being calm or composed). Similarly, after examining the sources of confusion during the MERS outbreak, Yang and Cho [3] reported that media content affected the public’s perception of MERS risk, and that this perception of a high level of risk affected the public’s reactions. Choi et al. [10] focused on the role of the media in shaping the public’s perceived risk of MERS. Based on online panel survey data during the MERS outbreak, they found that social media exposure was positively related to the formation of risk perceptions.

Some studies have focused on individuals’ responses and behaviors with regard to MERS. For instance, Kim et al. [11] performed a telephone survey involving 200 household members in and around Seoul, during the maturity stage of the outbreak of MERS in June 2015, Korea. They found that respondents who had low perceived risk of contracting MERS had low trust in the government’s ability to control MERS, and they generally held unfavorable attitudes toward quarantine. Moreover, Makhlouf et al. [8] reported that among 78 camel owners, the majority (74%) felt that they were protected from the disease, mostly because their farms were clean (78.1%) or because they had had a long history of working with camels without catching the disease (69.9%). Further, most respondents believed that washing hands with soap and water (84.9%) and keeping away from sick individuals (78.1%) were protective measures against MERS.

Our study focused on the factors that influence the perceived risk of MERS. A few previous studies have examined the determinants of the perceived risk of MERS. For example, Yang and Cho [3] reported that risk perception was positively associated with overreaction by the public (odds ratio: 2.80; 95% confidence interval: 2.17–3.60; *p* < 0.001). Moreover, Lee et al. [7] reported that the infection sensitivity of community residents during the MERS outbreak in Korea correlated with gender, age, occupation, and health behaviors. Based on a survey of male high-school students, Choi et al. [12] revealed that their risk perception score was 1.8 on a 4-point scale. Moreover, there was a positive correlation between knowledge, risk perception, and health behaviors. Additionally, knowledge and risk perception explained 15.1% of the variance in health behaviors, indicating that higher knowledge and higher risk perception were associated with higher protective behavior. With a more integrated causal model, Kim and Song [11] examined the causal relationships among risk characteristics, trust, risk perception, and preventive behavioral intention during the MERS risk/crisis situation. They found that risk characteristics were positively associated with risk perception. Moreover, trust was negatively associated with risk perception, but positively related to preventive behavioral intention.

While these previous studies focused on the perception factor, they ignored important factors such as value and resources. Therefore, we adopted a more comprehensive model to reflect all three factors, i.e., perception, value, and resources.

### 2.2. Determinants of Risk Perception

Among the three competing factors that explain risk perception, value pertains to the fundamental orientation of individuals, perception focuses on the cognitive factor in determining risk, and resources focuses on the objective state of individuals or the assets they possess.

#### 2.2.1. Value Factor

Value provides a basic orientation that directs perception and attitude [13]. Rokeach [14] defined a value as “an enduring belief that a specific mode of conduct is personally or socially preferable to an opposite or converse mode of conduct” (p. 5). Kim and Kim [13] suggested the value-based model for the judgment of science and technology. According to this model, value, as a fundamental set of factors, critically influences the perception of science and technology, i.e., perceived benefit, perceived risk, trust, negative feeling, and knowledge. Among various values, we focused on cultural value, science and technology optimism (referred to as “S&T optimism” henceforth), and ideology.

Cultural value: In risk studies, Douglas and Wildavsky [15] introduced the concept of cultural value, i.e., cultural bias, to explain risk perception. Based on the grid-group model, they argued that four cultural values (Egalitarianism, Individualism, Hierarchy, and Fatalism) influence risk perception. Moreover, in empirical testing, Wildavsky and Dake [16] discovered that egalitarians (who fear social deviance less than hierarchists and individualists do) fear technology to a great extent.

Among cultural biases, the present study focused on the impact of egalitarianism and individualism. According to the empirical studies of Brenot et al. [17], correlations between cultural biases and perceptions of 20 social and environmental risks were very weak, explaining 6% of the variance in risk perception. In particular, egalitarianism was correlated with a higher perception of risk. Moreover, Kim and Kim [18] showed that egalitarian social groups displayed higher perceived risk regarding a newly-developed information system, whereas individualistic groups revealed lower risk perceptions in this regard. Based on a meta-analysis, Xue et al. [19] reported that the overall weighted average effect size (r) for the association between egalitarianism and environmental risk perception was 0.25 (*p* < 0.001), whereas that for individualism was smaller (r = −0.17, *p* < 0.001) and in the opposite direction. Moreover, according to Marris et al. [20], egalitarians are predicted to be more concerned about large-scale environmental risks with potentially catastrophic consequences such as nuclear power and ozone depletion, whereas individualists would consider these risks to have been exaggerated. Based on these previous studies, we propose the following hypothesis:

**Hypothesis** **1** **(H1).** Egalitarianism has higher risk perception of MERS than individualism does.

S&T (Science and technology) optimism: S&T optimism represents a positive orientation toward science and technology and their byproducts. Cobb and Macoubrie [21] reported that respondents’ views of science influenced their risk and benefit perception related to nanotechnology. They showed that if individuals had positive beliefs about science (e.g., science solves the environmental problems), nanotechnology was regarded as more beneficial than risky. Additionally, Kim and Kim [13] reported that the value factor plays a key role in determining the perception factor. In particular, S&T optimism has a negative impact on perceived risk but a positive impact on other variables.

An optimistic attitude toward technology has an impact on the perceived benefit and risk of Genetically Modified Organism (GMO) products [22]. A study by Mohr et al. [23] revealed that pro-science and technology attitudes increase the acceptance of technology innovation in terms of social benefit and indulgence. Moreover, Costa-Font et al. [24] empirically showed that optimism decreased risk perceptions regarding, for example, climate change and GMO food.

Why are views about S&T related to risk judgment? According to the psychometric paradigm, Fischhoff et al. [25] proposed that nine risk characteristics influence perceived and acceptable risk. Among them, two characteristics, i.e., known to the science, and controllability, are critical factors in risk judgment. Thus, if individuals feel that they do not precisely understand the risk object or if they do not fully believe in its controllability, they exhibit higher risk perception and less acceptance. Considering this logic, science and technology are a means for acquiring precise knowledge and for controlling an unknown risk. This acquisition of precise knowledge and improved controllability of risk usually reduces the uncertainty that increases risk perception. According to Siegrist et al. [26], the general confidence that everything is under control negatively influences risk perception. In this vein, positive belief in science and technology might negatively influence the perceived risk of MERS. Moreover, trust in science and scientists, i.e., an alternative version of optimism, has an impact on risk perception. For example, according to Hmielowski et al. [27], trust in scientists is an important instrument that many individuals use when reporting their opinions on science-related topics; the higher the trust, the lower the perceived risk. Considering these findings, we propose the following hypothesis:

**Hypothesis** **2** **(H2).** Strong S&T optimism in individuals decreases the perceived risk of MERS.

Ideology: Ideology acts as an anchor for risk judgment. According to Mayer et al. [28], progressive individuals show greater sensitivity to risk than conservative individuals do. According to McCright [29], political conservatives and those with higher incomes and more education perceive less threat from global warming than their counterparts do. Additionally, they found that conservative white men exhibit a higher tendency to express skeptic beliefs in climate change. Further, as expected, political conservatism (vs. liberalism) was related to perceiving greater risk of voluntary hazards to the self and perceiving less risk for documented collective risks [29]. This evidence suggested that progressive individuals perceive more risk from specific events than conservative individuals do.

Moreover, when MERS broke out, the ideological divide between political parties, not the public, provided the critical context for the important role of ideology in risk perception. The MERS outbreak increased the distrust toward the right-oriented President Park Geun-hye. The conflicts between the right and the left peaked when the left-oriented mayor of Seoul, Park Won-soon, criticized the president. Mayor Park publicly blamed the central government’s failure to filter suspected MERS patients and for hiding important information that could have been useful for the public and municipalities. President Park, however, criticized the mayor, saying that the Seoul Mayor’s independent actions could lead to confusion among the public. The conservative ruling party accused the mayor of spreading false rumors [30]. Such political conflicts started just not from the MERS case. The Sewol ferry disaster that occurred 2014 and killed more than 300 individuals revealed similar ideological conflicts, as like MERS case. According to Jin and Song [31], the disaster response in the case of the Sewol ferry disaster was unsuccessful because the Korea Coast Guard (KCG) was more concerned about hierarchical, political, and legal accountability than about professional accountability during the accident.

In short, South Koreans tend to consider the conflict over the outbreak of MERS as an ideological issue and not a health issue. Therefore, the impact of individuals’ ideological position on their perceived risk needs to be examined. Therefore, the following hypothesis was proposed:

**Hypothesis** **3** **(H3).** Progressive individuals have higher risk perception than conservative individuals do.

#### 2.2.2. Perception Factor

The psychometric paradigm focuses on what kinds of risk characteristics influence individuals’ perceptions regarding different hazards. This paradigm regards risk as having subjective rather than objective attributes. According to Slovic et al. [32], this paradigm uses psychological scaling to produce cognitive maps of risk attitudes and perceptions. Accordingly, it is suggested that individuals make quantitative judgments about the current and desired riskiness about diverse hazards and the desired level of regulation about them. These judgments are then related to the judgments about other properties, including (1) the hazard’s characteristics, such as voluntariness, dread, knowledge, and controllability; (2) the benefit provided by the hazard; and (3) the number and seriousness of the deaths caused by the hazard [32]. Within the psychometric paradigm, we focused on the four variables described in the following sections, because they have frequently been analyzed in risk perception studies pertaining to epidemic diseases [3,33,34].

Trust: Risk studies have frequently examined trust as the target variable. For instance, Siegrist et al. [35] empirically showed that social trust has an indirect effect on the emotional aspect that influences perceived benefits and risks related to food involving nanotechnology. Moreover, Kim and Kim [13] showed that trust increases the acceptance of new and emerging technologies. Such findings imply that trust plays a critical role in attenuating the degree of perceived risk. Based on survey data analysis, Yang and Cho [3] reported that trust in the media (broadcasting), local government, and NGOs was positively associated with cumulative risk perception, whereas trust in the medical profession, the central government of South Korea, health policy, and the society was negatively associated with overall risk perception. Kim and Song [11] demonstrated that trust is negatively associated with risk perception but positively related to preventive behavioral intention. Further, Kim et al. [36] reported that when respondents had low trust in the government’s ability to control MERS, they generally held unfavorable attitudes toward quarantine.

Recently, several studies have reported the contingent effect of trust. According to Siegrist and Cvetkovich [35], there was a strong correlation between social trust and judged risks/benefits with regard to hazards when individuals did not possess much knowledge. Thus, they showed that the impact of trust depends on the amount of knowledge. To measure social trust, Seigrist and Cvetkovich [35] examined whether respondents agreed or disagreed with the statement that they “trust in authorities regulating each activity or technology”. Results showed strong correlations between social trust and perceived risk/benefit of hazards when individuals did not possess much knowledge. Thus, trust decreased the perceived risk and increased the perceived benefit. In this context, the following hypothesis was proposed:

**Hypothesis** **4** **(H4).** The greater the amounts of trust individuals have will decrease the perceived risk of MERS.

Perceived vulnerability: Perceived vulnerability is a subjective perception about how weak individuals feel when faced with specific threatening factors. It comprises the following four basic elements of the health belief model: (a) vulnerability to a negative event; (b) severity of a negative event; (c) benefits of specific preventive actions; and (d) barriers to performing preventive actions [37] (p. 390). Gerrard et al. [37] explained that perceived personal vulnerability is usually depicted as a necessary (but insufficient) motivator of precautionary behavior. In the case of disease, perceived vulnerability refers to the extent to which individuals feel that they would acquire some disease. According to Brug et al. [34], not only the perceived ability to avoid SARS, but also one’s ability compared to others, influences the perceived risk of acquiring SARS and the worry about contracting the disease. In an empirical study, Tang and Wong [33] found that elderly participants who perceived greater personal vulnerability to SARS were more likely to adopt SARS-preventive behaviors. Based on these findings, the following hypothesis was proposed:

**Hypothesis** **5** **(H5).** Individuals holding a higher vulnerability have an increased risk perception of MERS.

Belief in resilience: Resilience has various meanings. Holling [38] described resilience as “a measure of the persistence of systems and of their ability to absorb change and disturbance and still maintain the same relationships between populations or state variables” (p. 14). Disaster resilience is the ability of individuals, communities, organizations, and states to adapt to and recover from hazards, shocks, or stresses without compromising long-term prospects for development [39]. In the present study, we focused on the subjective resilience that individuals constructed. According to the Hyogo Framework for Action [39], those who strongly believe in their own resilience tend to exhibit less risk perception because they have a great deal of confidence in being able to recover from a byproduct of a hazard afterward. Thus, the following hypothesis was proposed:

**Hypothesis** **6** **(H6).** The strong belief in resilience lowers individuals’ risk perception of MERS.

Self-efficacy: Self-efficacy refers to an individual’s perception of her or his competence at successfully performing a behavior [40]. Tang and Wong [33] reported that elderly participants who possessed greater self-efficacy were more likely to adopt the suggested preventive behaviors for SARS. Moreover, Choi et al. [8] explained that individuals with higher self-efficacy have lower risk perceptions regarding a health issue, whereas those with lower self-efficacy have higher risk perceptions regarding the same health issue. They also demonstrated that self-efficacy for MERS was negatively associated with risk perceptions regarding MERS. Moreover, Choi et al. [10] reported that self-efficacy was found to moderate the impact of social media on risk perceptions regarding MERS. Makhlouf et al. [8] found that, since 60% of camel owners had a high self-efficacy score, a very low percentage of them were using protective measures (4–12%). These findings informed the following hypothesis:

**Hypothesis** **7** **(H7).** Lower self-efficacy increases individuals’ risk perception about MERS.

#### 2.2.3. Resource Factor

Benford et al. [41] suggested the importance of resources in risk judgment. They revealed that minorities exhibited more perceived risk because they lacked resources and alternatives that provided a kind of shelter or buffer from risk. Therefore, our study focused on the following three resources: (1) economic resources, i.e., income and social class; (2) health resources, i.e., perceived health state in our research; and (3) knowledge.

Economic Resources: Economic resources provide various instruments to avoid a hazard, e.g., more safe places and speedy vehicles, which can be used to escape from disease. Income and social class represent economic resources. Huang et al. [42] reported that public acceptance of nuclear power decreased significantly after the Fukushima nuclear accident. Additionally, they found that those with lower income were the most sensitive group, who exhibited a decrease in the support for nuclear power. Another study revealed that individuals in upper-social-class tended to perceive a lower risk than those in lower-social class did [43]. According to Kim and Kim [13], higher social class reduces the perceived risk about nanotechnology and animal cloning. Based on these findings, we purpose the following hypothesis:

**Hypothesis** **8** **(H8).** Higher income or social class decrease the perceived risk of MERS.

Health Resource: Health is a personal resource and a means for protecting one’s life in terms of both objective physical and subjective states. According to the health belief model, individuals who are susceptible to a health problem tend to exhibit behaviors to reduce their risk from the health problem. According to Ko [44], a subjective health state influences risk perception. He reported that self-perceived poor health is related to risk perception of cardiovascular disease (CVD). Sadique et al. [45] found that the health that individuals maintain negatively influences their precautionary behavior for a hypothetical influenza pandemic. Further, they reported that those who are healthier do not avoid entertainment, limit shopping, take absence from work, keep children from school, limit contact with family and friends, avoid seeing the doctor, or stay indoors. Thus, the following hypothesis was proposed:

**Hypothesis** **9** **(H9).** Individuals with more health resources are likely to perceive risk less than those with less health resources are.

Perceived Knowledge: Social studies of science have stressed on the role of knowledge in making judgments about science and technology. The deficit model is the most well-known model for explaining the role of knowledge in public judgment. According to Dickson [46], the deficit model argues that a lack of adequate knowledge about science mainly influences public skepticism toward modern science and technology. Therefore, if provided sufficient information about science and technology to overcome this lack of knowledge, the public could change its mind and make a good decision.

With reference to knowledge, based on an online survey performed after the epidemic had peaked, Yang and Cho [3] reported that students had 53.5% of the essential knowledge pertaining to MERS. Similarly, based on a cross-sectional study, Coulibaly et al. [47] reported that 255 (55.4%) respondents had knowledge of MERS-CoV. Choi et al. [10] found a positive correlation between knowledge/risk perception and health behaviors. Furthermore, Makhlouf et al. [8] reported that majority of the camel owners (79.5%) who participated in their study had a low to moderate level of knowledge of MERS-COV. More than half of them had a low perceived risk of susceptibility to contract the disease. Choi et al. [10] reported that the proportion of correct answers to questions testing MERS-related knowledge was 71.6% among male high-school students.

Cobb and Macoubrie [21] showed that greater knowledge influences the risk judgment about technology. For example, those who are more knowledgeable tend to believe that the benefits from nanotechnology would be more than the risks. Rolison and Hanoch [48] found that respondents who were more knowledgeable about Ebola, perceived less risk of contracting the virus and were less worried about the virus, but they also regarded Ebola as more serious than less knowledgeable respondents did. According to Abbate et al. [49], knowledge of preventive measures has a positive impact on the perception of risk for avian influenza; however, general knowledge of avian influenza does not explain the preventive measure behaviors. Considering these findings, we proposed the following hypothesis:

**Hypothesis** **10** **(H10).** More knowledge related to MERS decreases individuals’ perceived risk of MERS.

**Figure 2:**
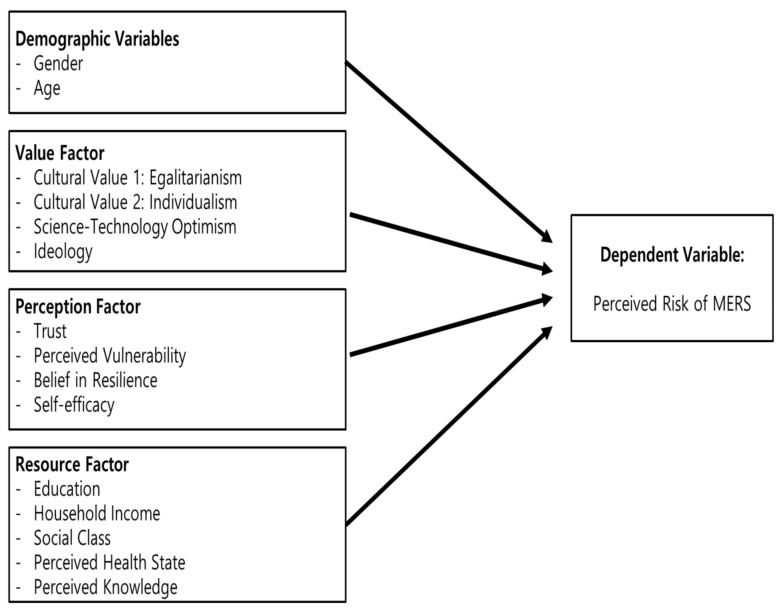
Research framework.

Based on various variables comprising the three factors examined in the present study (perception, value, and resources), we developed the research model shown in Figure 2. We hypothesized that not only the perception factor, but also the value and resource factors, influence the perceived risk of MERS.

## 3. Sample and Measure

### 3.1. Sample 

Our analysis was based on survey data that measured the attitude and behaviors towards risk and communication related to science and technology. The survey was carried out from June to September 2015, using an interviewer-administrated questionnaire. Participants were selected in three stages. First, we categorized the respondents according to gender and age. Second, we classified them again according to social groups, such as students, housemaids, salaried men, and elder organization. Finally, we randomly selected the respondents from those social groups. We collected the data from 1000 respondents. However, we used the data collected from 814 respondents for the analysis because we excluded incomplete or inaccurately answered questionnaires. Table 1 shows the characteristics of the respondents and mean, S.D. of perceived risk of MERS.

In terms of education level, 1.9% of the respondents were below middle school level, 21.7% were high school graduates, 40.4% were college student, 33.3% were university graduate, and 3.1% were a graduate of graduate school. With reference to gender, 47.8% of the participants were males and 52.2% were females. Regarding age, 52.5% of the respondents were aged below 29 years, 14.0% and 20.0% were in 30s and 40s, respectively, and 13.5% were in 50s or above. The unit of income was the Korean won. Further, 19.7% of the participants had an income less than 2.9 million won, while 27.9%, 29.2%, and 23.2% had incomes from 3 million won to 4.99 one, from 5 million won to 5.99 one, and more than 7 million won, respectively. Overall, the present sample comprised more college graduates and young respondents.

### 3.2. Measure

The main measures used in this study are presented in Table 2. To ensure more validated measures, we adopted the measures used in previous studies. To examine several variables that measure one concept, we used the mean values of them. To measure the reliability of the measures, we calculated the Cronbach’s Alpha values, which were reported in the third column of Table 2. Ideology and social class were assessed using a ten-point scale (1 = left, 10 = right/1 = lowest social class, 10 highest one). Respondents choose one point on the ten-point scale based on their personal ideology and social class.

Among independent variables, the concept of trust is complicated and multi-dimensional, and it varies across actors, attributes, components, and levels. First, actors are the targets of trust. The government, scientists, and NGOs have frequently been examined as objects of trust. In terms of actor-centered approaches, Yang and Tang [50] identified the following 3 types of trust: trust in administrative institution, trust in legal institution, and trust in societal institution. Second, the concept of trust depends on the level of trust, i.e., individual versus institutional trust. Rousseau et al. [51] defined individual trust as “a psychological state comprising the intention to accept vulnerability based upon positive expectations of the intentions or behavior of another” (p. 395). In the other hand, Moy and Pfau [52] defined institutional trust as the positive orientation toward a political system. Third, characteristics of trust are one of the standards to distinguish various forms of trust. In this regard, Rousseau et al. [51] suggested two types of trust, relational and calculative. While the former refers to the relationship between the trusting person and the other, the latter pertains to the past behavior of the other and/or constraints on future behavior.

These dimensions of trust informed the concept of trust that was adopted in the present study. The measures of trust used in our study referred to experts and the government as actors, used trustworthiness as an attribute of trust, and examined trust perception at the individual level. Therefore, we assumed that more credible actors could increase the individual perception of trust.

Also, we measured vulnerability for other diseases, not MERS, because if we had measured the vulnerability of MERS, there would have been problems of its higher correlation with perceived risk. Indeed, there are several common attributes between the vulnerability and risk perception of MERS.

We adopt the measure of cultural biases from Dake [53], Dake [54], and Marris et al. [20], Resilience from Jew et al. [55], self-efficacy from Choi et al. [8]. All measures used a five-point Likert scale (1 = strongly disagree, 5 = strongly agree) except vulnerability.

## 4. Analysis

### 4.1. Basic Description

We calculated Pearson’s correlation coefficients to examine the relationships among variables. These results have been presented in Table 3.

The perceived risk of MERS had significant positive relationships with age, egalitarianism, individualism, S&T optimism, perceived vulnerability, belief in resilience, self-efficacy, knowledge, and social classes, whereas it had negative relationships with trust and education. Among the correlation coefficients, trust had the highest correlation with perceived risk of MERS, followed by knowledge, individualism, vulnerability, and self-efficacy. These results imply that trust is a key factor in reducing perceived risk of an epidemic disease. Moreover, three of five variables that showed high correlations with perceived risk were related to the perception factor. This implied that perception, not value and resource, plays a critical role in explaining perceived risk.

Three more interesting findings were observed from the correlation analysis. First, knowledge was found to have a significant relationship with perceived risk, whereas educational level was not. This indicates that officially or formally obtained information based on the educational level can be distinguished from unofficially or informally obtained information through personal efforts. Such a plausible argument of distinctive relationship between educational level and knowledge is supported by the lower correlation coefficient (0.005) in Table 3, not being significant. Second, among economic resources, higher social class (measured by respondents’ judgment of their own state of social class on a 10-point scale, i.e., 1 = lower class, 10 = high class) had a significant impact on perceived risk, whereas household income did not. This implies that subjective wealth is more important than objective wealth is. Third, perceived health state did not have a significant relationship with perceived risk. Generally, good health is regarded to lead to a positive belief in the recovery from diseases [44,45]. However, our analysis did not confirm the positive role of good health.

### 4.2. Causal Structure

To understand the impact of the perception, value, and resource factors on perceived risk, we regressed the perceived risk of MERS on sixteen predictors. Table 4 shows the results of the regression analysis. We controlled for three socio-demographic variables; age, gender, and education. Additionally, to understand the effect of ideology on perceived risk, we set the neutral group as a reference.

We checked the potential multicollinearity issue by examining tolerance and Variation Inflation Factor (VIF). Our data showed that the values of tolerance (0.712 to 0.946) were beyond 0.1 and the VIFs (from 1.057 to 1.404) did not cross 10. Therefore, there was no issue with multicollinearity.

As is evident from Table 4, demographic variables (gender, age) do not have a significant impact on the perceived risk of MERS. This finding needs to be discussed further, because it is contrary to the general belief and previous findings. For example, it has been generally accepted that older individuals are more sensitive to a disease than younger individuals are, because the former are more vulnerable than the latter. Yang and Cho [3] reported that the elderly obtained lower scores on risk perception (concern over contracting MERS through indirect contact) (β = 0.07; *p* = 0.004).

Moreover, contrary to the present findings, females are considered to exhibit higher risk perception compared to males, as evidenced by the findings of Yang and Cho [3], who reported that women had a higher risk perception than men did. Moreover, according to Choi et al.’s [8] study on MERS, gender had a positive relationship with risk perception (β = 0.09, *p* < 0.01). According to Kim and Kim [13], education level was related to lower perceived risk with regard to nanotechnology and animal cloning. Our analysis did not confirm such a negative impact of education.

Concerning the value factor, the two cultural biases (egalitarianism and individualism) and S&T optimism did not reveal significant coefficients. Such results suggest that value has no relationship with risk perception of infectious diseases, which implies that the vital need for survival overrides the existing value that individuals have. In other words, the perceived risk of a disease might be related to the outer layers of the thinking system, wherein value lies at the core and perception/cognition is at the peripheral.

With reference to the perception factor, trust had the strongest power in explaining the perceived risk of MERS. Our results confirmed Yang and Cho’s [3] finding that trust in media was positively associated with risk perception (*p* < 0.001). Moreover, Kim and Song [11] reported that trust had a negative impact on risk perception. Moreover, those with higher trust showed more interest in preventative behaviors.

The vulnerability that individuals felt toward MERS significantly influenced their perceived risk of MERS. According to Gerrard et al. [37], the perception of vulnerability is weakly but significantly related to risk behavior, suggesting that—to a limited extent—individuals do take their behavior into account when estimating their vulnerability to HIV.

Belief in resilience and self-efficacy did not have a significant impact on the risk perception in the present study. Although the measures of belief resilience (“The problem of MERS can be overcome by human effort”, “The problem of MERS can be solved by scientific and technological efforts”, “I can certainly overcome MERS if I acquire it”, “If I acquire MERS, I can be cured more easily than others can”) and self-efficacy (“I can certainly overcome MERS if I acquire it”, “If I acquire MERS, I can be cured more easily than others can”) connoted individuals’ self-confidence in overcoming MERS, they did not have a significant explanatory power. MERS is one of the most serious epidemic diseases, which, if infected, is difficult to overcome. Therefore, such irresistible attributes of MERS may have influenced the ineffective role of resilience and self-efficacy observed in the present study.

Finally, among the variables in the resource factor, household income and social class did not demonstrate a significant role in predicting the perceived risk of MERS. This finding is in contrast with Choi et al.’s [10] finding that respondents with higher levels of income tended to perceive more risk than those with lower levels of income did.

Perceived health state of individuals negatively influenced their perceived risk. Specifically, those with good health revealed less perceived risk than those with poor health did. This finding contrasted with the present results of the correlation analysis, which did not reveal a significant correlation between perceived health and perceived risk.

Moreover, we found that the higher the knowledge of individuals, the higher their perceived risk of MERS. Similarly, Choi et al. [12] found a positive relationship between knowledge and risk perception. However, these findings contradicted Yang and Cho’s [3] findings in which the level of knowledge about MERS was not associated with risk perception.

To compare the explanatory power of the three factors, we calculated the F-value and R^2^ in each model. The last four lows show the analysis results. The perception factor explained 31.3% of the variance in perceived risk, while the other two factors had little effect, each having 4.9% and 5.7%. These findings imply that perception is more important than value and resource.

### 4.3. Discussion

Our study showed, first, that the perception factor explained a larger proportion of the variance in perceived risk as compared to the value and resource factors. Further, among the variables of the perception factor, trust was a key component for alleviating the fears related to a pandemic disease such as MERS. Trust is an important variable because it plays a various significant roles. For example, individuals tend to use social trust to complement lack of knowledge. Siegrist and Cvetkovich [35] showed that when there was limited knowledge, trust played an important role in judging emerging technologies. Our analysis showed that not only trust, but also knowledge had an impact on the perceived risk of MERS. This means that though trust may stem from the lack of knowledge, it plays a significant role in risk perception. Kim et al. [30] argued that when MERS broke out, the government did not disclose information about the hospital that reported the first MERS patient. In such a situation, with no information and knowledge, distrust plays a critical role in risk judgment.

Second, less vulnerability was found to increase perceived risk. This finding suggests that more appropriate approaches are needed to focus on more vulnerable and less healthy groups. Thus, special programs need to provide such groups a feeling of relief and safety by mobilizing precautionary strategies. Vulnerable groups such as the elderly, younger individuals, those with a disability, and females have been found to exhibit higher perceived risk. Therefore, more tailored strategies need to be implemented to make them feel safe.

Third, among the variables of the resource factor, higher knowledge was found to be related to higher perceived risk. It is generally observed that more knowledge generally reduces perceived risk. However, in MERS, knowledge was found to amplify perceived risk. Thus, it infers that the effect of knowledge seems to vary according to the context. Therefore, better knowledge management strategies need to be implemented. This critical role of knowledge suggests that the government and other actors should mobilize risk communication during an epidemic to provide appropriate and precise information. In this regard, Yang and Cho [3] reported that risk perception was associated with the information delivered by the media. Therefore, providing accurate information and data to the public will be important in managing future crises. Song et al. [9] suggested that, in the case of a breakout of a novel and highly contagious disease such as MERS, the government must deploy a response system that includes the provision and dissemination of reliable information and inhibits the online diffusion of false information. Thus, to ensure appropriate knowledge management, disease control authorities should develop an effective method of retrieving, sharing, and storing information related to the disease.

## 5. Conclusions

### 5.1. Summary

Our study aimed to analyze the determinants of perceived risk of MERS in Korea. To this end, we examined a more comprehensive model that focused on three factors such as value, perception, and resource.

Based on the survey data, we identified the significant role of perception and resources; however, the role of value was not confirmed. In the perception factor, trust decreased perceived risk, whereas perceived vulnerability increased it. In the resource factor, maintaining a good health contributed to reducing perceived risk, whereas more knowledge amplified the perceived risk of MERS. The value factor did not explain the risk perception. Additionally, among the variables examined in the present studies, trust explained the largest proportion of the variance in perceived risk, followed by perceived health state, knowledge, and vulnerability. Our findings suggest that, with reference to infectious diseases, perception and resource are key factors that determine risk perception. Moreover, the perception factor is more important than the resource one.

### 5.2. Implications 

It is important to note that, among the independent variables, trust explained the largest proportion of variance in the perceived risk of MERS. This finding suggests that trust management by the government or other agencies is important in decreasing the perceived risk of an infectious disease. To increase the trust of individuals, the government needs to pay attention to effective communication with the public. According to the WHO outbreak communication guidelines, the following five key points need to be focused on during outbreak communication: (1) build, maintain, or restore trust; (2) announce early; (3) be transparent; (4) understand the public; and (5) incorporate risk communication into preparedness planning. When emerging infectious diseases break out, it is important to mobilize the media to make effective risk communication and to ensure public safety [56]. Likewise, on examining the MERS outbreak in Korea, Lee [57] suggested that we need several categories of successful crisis management communication, such as rapidness, consistency, openness, existence of control tower, and use of a manual. These suggestions stress on the role of effective communication strategies by the government.

Thus, more organized management strategies are required for building the trust. In this regard, as mentioned in the theoretical background Section 2, it is necessary to understand the multidimensional structure of trust. Trust varies according to the actors, attributes, and level of units. Therefore, the accountable controller during an epidemic needs to develop more diverse strategies to cover different forms of trust.

Our study has some limitations. Yang and Cho [3] showed that media content affects the public’s perception of MERS risk and that the higher perceived risk leads to overreaction. However, our analysis did not consider such media effects. Additionally, since there were very few studies on the perceived risk of MERS, it is difficult to compare our findings to previous ones. In the future, more empirical studies are needed to verify our findings. Moreover, four cultural types should be included in the theoretical model. However, we did not include hierarchism and fatalism in our research model because of the low level of reliability of the measures for these two cultural biases. However, considering that these two cultural types might be related with the public’s health-related perceptions (e.g., Kahan et al. [58] 2010; Song [59]; Song et al. [60]), their exclusion is a limitation of the present study.

## Figures and Tables

**Table 1:** Distribution of respondents.

Category		Number (%)	Mean of Perceived Risk of MERS	S.D. of Perceived Risk of MERS
Education	Less than middle school diploma	16 (1.9%)	2.938	0.605
	High school diploma	174 (21.7%)	3.056	0.634
	Undergraduate	321 (40.0%)	2.948	0.606
	University graduate	267 (33.3%)	2.979	0.612
	Graduate school	25 (3.1%)	2.920	0.866
	Total	803 (100.0%)	2.981	0.623
Gender	Male	387 (47.8%)	3.019	0.612
	Female	423 (52.2%)	2.953	0.633
	Total	810 (100.0%)	2.984	0.624
Age (years)	Less than 29	425 (52.5%)	2.955	0.586
	30–39	113 (14.0%)	2.940	0.653
	40–49	162 (20.0%)	2.978	0.641
	over 50	109 (13.5%)	3.155	0.690
	Total	809 (100.0%)	2.984	0.624
Household Income(Million won)	≤299	125 (19.7%)	2.950	0.614
	300–499	177 (27.9%)	3.003	0.629
	500–699	185 (29.2%)	2.999	0.589
	≥700	147 (23.2%)	3.021	0.661
	Total	634 (100.0%)	2.996	0.621

Note: Mean, S.D. is about perceived risk of MERS.

**Table 2:** Measures and reliability of dependent/independent variables.

Concept	Measure	Reliability (Cronbach Alpa)
Perceived risk	-Compared to other diseases, MERS is the most dangerous	0.593
	-MERS is a serious threat to me and my family	
	-The MERS is serious risk that threatens the survival of humans	
	-I have a high possibility of contracting MERS	
	-Compared to others, I am highly likely to contract MERS	
Egalitarianism	-We need an overall reform for the equal distribution of wealth	0.610
	-Making the society equal will solve many problems	
	-Equity is the best solution to all problems	
Individualism	-Poverty faced by individuals is caused due to their less efforts	0.654
	-A competitive society is better	
	-It is natural that only those who are stronger and competent survive	
S&T (Science and technology) optimism	-Science and technology makes our life healthier and convenient	0.661
	-Due to science and technology, the earth will not face the exhaustion of resources	
	-Science and technology will solve all problems	
	-Science and technology provides the future generation more opportunities	
Trust	-I do not at all believe the experts’ explanation about MERS (R)	0.624
	-I think that all of rumors related with MERS are true (R)	
	-I think that the rumors about MERS that are circulating on the Internet are true (R)	
	-I think that the facts that the government argued to be false are actually true (R)	
(Perceived) Vulnerability	-Considering your health condition, to what extent do you think you will acquire the following diseases (each) within the next one or two years? (1) heart disease; (2) Tuberculosis; (3) diabetes (1 = Least likely to acquire, 5 = Most likely to acquire)	0.873
(Belief in) Resilience	-The problem of MERS will be overcome by human effort	0.752
	-The problem of MERS will be solved by scientific and technological efforts	
Self-Efficacy	-I can certainly overcome MERS if I acquire it	0.398
	-If I acquire MERS, I can be cured more easily than others can	
	-MERS is a disease I can avoid if I am careful	
(Perceived) Health State	-My health is good	0.867
	-I keep good health	
Perceived Knowledge	-For the sake of precaution against MERS, I seek much information related to it	0.755
	-I have much more knowledge about MERS than others do	

Note: S.D.: Standard Deviation.

**Table 3:** Pearson’s correlation coefficients between perceived risk and other variables.

Variables	1	2	3	4	5	6	7	8	9	10	11	12	13	14	15
1. Perceived Risk of MERS	1														
2. Gender	−0.053	1													
3. Age	0.082 **	−0.052	1												
4. Ideology (Progressive)	0.064	−0.004	−0.07 *	1											
5. Egalitarianism	0.087 **	−0.03	0.032	0.077 *	1										
6. Individualism	0.181 ***	−0.098 ***	0.031	−0.101 **	0.177 ***	1									
7. S&T Optimism	0.092 ***	−0.134 ***	0.102 ***	−0.005	0.088 **	0.097 ***	1								
8. Trust	−0.538 ***	0.015	−0.049	−0.097 **	−0.137 ***	−0.167 ***	−0.056	1							
9. Perceived Vulnerability	0.156 ***	−0.033	0.023	−0.021	0.05	0.073 **	0.007	−0.059 *	1						
10. Belief in Resilience	0.059 *	−0.034	0.111 ***	0.054	0.094 ***	0.091 ***	0.066 *	−0.032	−0.156 ***	1					
11. Self-efficacy	0.154 ***	−0.021	0.090 *	0.051	0.111 ***	0.183 ***	0.076 **	−0.159 ***	−0.119 ***	0.441 ***	1				
12. Perceived Knowledge	0.255 ***	0.028	0.043	0.098 **	−0.027	0.032	0.04	−0.270 ***	0.122 ***	0.012	0.152 ***	1			
13. Education	−0.058 *	−0.048	−0.454 ***	0.087 **	−0.057	−0.070 **	−0.064 *	0.042	0.002	−0.044	−0.054	0.005	1		
14. H. Income	0.032	−0.100 *	0.022	0.099 **	0.074 *	0.046	0.033	−0.027	−0.048	0.035	0.006	−0.037	0.118 ***	1	
15. Social Class	0.071 *	0.041	0.035	0.184 ***	−0.027	0.059	0.05	−0.127 ***	−0.013	−0.02	0.002	0.102 ***	0.097 **	0.259 ***	1
16. Perceived Health State	0.019	−0.066 *	0.018	0.03	0.009	0.075 **	0.052	−0.141 ***	−0.342 ***	0.326 ***	0.358 ***	0.088 **	−0.02	0.065	0.084 **

* *p* < 0.1; ** *p* < 0.05; *** *p* < 0.01.

**Table 4:** Regression analysis: Perceived risk of MERS as dependent variable.

Factor	Variables	B (Unstandardized Coefficients)	SE (Standard Error)	Beta
F1: Socio-demographic Factors	Constant	4.263	0.39	
	Gender (1 = female)	−0.051	0.047	−0.041
	Age	0.001	0.002	0.008
	Education level	−0.002	0.058	−0.002
F2: Value Factors	Egalitarianism	−0.04	0.033	−0.046
	Individualism	0.045	0.032	0.055
	S&T Optimism	0.043	0.036	0.044
	Ideology 1 (Conservative)	0.017	0.089	0.008
	Ideology 2 (Progressive)	−0.099	0.063	−0.059
F3: Perception Factors	Trust	−0.526 ***	0.038	−0.545
	Perceived Vulnerability	0.064 **	0.03	0.085
	Belief in Resilience	0.024	0.033	0.031
	Self-efficacy	0.049	0.041	0.051
F4: Resource Factors	Household Income	0.012	0.039	0.012
	Social Class	−0.003	0.017	−0.007
	Perceived Health State	−0.113 ***	0.031	−0.158
	Perceived Knowledge	0.079 ***	0.029	0.109
F-Value		17.721 ***		
R^2^		0.378		
Ad. R^2^		0.356		
F1’s F-value/R^2^		0.009/3.677 **		
F2’s F-value/R^2^		0.049/6.540 ***		
F3’s F-value/R^2^		0.313/91.644 ***		
F4’s F-value/R^2^		0.057/6.349 ***		

* *p* < 0.1; ** *p* < 0.05; *** *p* < 0.01.
